# How much does the absence of the ‘hidden population’ from United Kingdom household surveys underestimate smoking prevalence?

**DOI:** 10.1111/add.70071

**Published:** 2025-05-02

**Authors:** Emma Beard, Lion Shahab, Jamie Brown, Sharon Cox

**Affiliations:** ^1^ Research Department of Behavioural Science and Health University College London London UK; ^2^ Department of Epidemiology and Public Health University College London UK; ^3^ Behavioural Research UK London UK

**Keywords:** hard‐to‐reach populations, hidden populations, household surveys, population surveys, sampling, smoking

## Abstract

**Background and aim:**

Sampling frames used by population surveys may result in an underestimation of smoking prevalence as those not residing in households, also known as the ‘hidden population’, are not sampled. This includes people living in care and residential homes, populations experiencing homelessness, as well as those who have an “absent or “temporary” household status (e.g. bed and breakfasts, unsupported temporary accommodation, and those in prison). This study estimated the impact of including these populations on smoking prevalence in the UK.

**Methods:**

Data from UK government reports and published studies were used to derive estimates of the size of the hidden populations and rates of smoking within these populations. The impact of including the ‘hidden population’ on overall smoking prevalence in the UK was estimated, along with a correction factor to account for their exclusion.

**Results:**

The hidden population over the age of 18 was estimated to be around 1.9 million with a smoking prevalence of between 58% and 66%. Accounting for this hidden population in smoking prevalence surveys may inflate estimates by a correction factor of 1.12 to 1.14. This means smoking prevalence in 2022 would increase from a reported 12.9% to an adjusted range of 14.5–14.8%.

**Conclusion:**

The absence of the ‘hidden population’ from smoking prevalence surveys leads to underestimation of smoking rates in the UK. Based on the estimated correction factor, and assuming smoking continues to decline at current rates, achieving the UK government‘s target of <5% by 2030 would be substantially delayed.

## INTRODUCTION

Smoking remains the single biggest preventable cause of death and illness in the United Kingdom (UK) [1]. In 2021, an estimated 76 thousand people died from smoking‐related causes in the United Kingdom [2]. Accurately measuring smoking prevalence is critical for addressing this major public health issue. The main survey for evaluating smoking prevalence in the United Kingdom is the Annual Population Survey (APS) by the Office for National Statistics (ONS) [3].

Household surveys, such as the APS, provide important information on smoking prevalence and patterns of public health problems. However, concerns have been raised about their representativeness of the entire population. This is in part because of the under‐recruitment of so‐called ‘hard‐to‐reach’ groups [4, 5]. These groups, including vulnerable migrants and sex workers, may be difficult to reach because of physical or social barriers. Weighting adjustments are used to account for non‐response among these groups, ensuring that participants reflect similar socio‐demographic characteristics as non‐respondents. Effective bias reduction relies on access to auxiliary information such as health indices and socio‐demographic data.

Another group, known as the ‘hidden population’, are entirely absent. This group lacks defined limits or sampling parameters, meaning there is no existing sampling frame for them. For instance, household surveys using probability sampling methods typically exclude individuals in non‐private households [6]. Non‐private households include communal establishments like care homes, long‐stay hospitals and some groups experiencing homelessness (such as those sleeping rough or those in temporary accommodations like bed and breakfasts). This category also encompasses communities like Gypsy, Roma and Travellers, as well as students in halls of residence [7, 8].

The exclusion of this ‘hidden population’ leads to non‐coverage or coverage error, where the sampling frame does not match the target population. This omission of ‘hidden populations’ poses significant challenges, particularly as smoking prevalence among the ‘hidden population’ can be 4 to 8 times higher than the general population. For instance, up to 80% of people who are experiencing homelessness or involved with the criminal justice system smoke [9, 10, 11]. To date, the lack of recognition of coverage problems when using estimates from household sample surveys stems partly from there having been no systematic attempt to estimate the size and distribution of the entire ‘hidden population’ [7]. As smoking rates continue to decline in the United Kingdom, the proportion of smokers from ‘hidden populations’ will grow larger and finite resources will need to be targeted. However, targeted investment cannot be achieved without better estimates of smoking rates within these groups and understanding how their omission affects overall smoking rates.

One pragmatic and cost‐effective approach to address the exclusion of the ‘hidden population’ from household surveys is deriving and applying a correction factor. This method involves adjusting collected survey data to account for the estimated ‘hidden population’. To ensure statistical robustness, the correction factor can also be applied to confidence intervals and must be regularly updated to maintain accuracy and relevance. Of course, the effectiveness of the correction factor approach is highly dependent on the quality and availability of auxiliary data.

Therefore, the objectives of this article are:

1. To estimate the size of the ‘hidden population’ excluded from household surveys.
2. To determine the potential impact of excluding the ‘hidden population’ on smoking prevalence estimates in the United Kingdom.
3. To derive and apply a correction factor to adjust smoking prevalence estimates.

For objectives 2 and 3, the APS estimates from 2022 will be used as a baseline. This is because it is the primary source of data used by the UK government to estimate smoking prevalence and it uses rigorous sampling and data collection methods. Incorporating a correction factor into such a methodologically sound survey enhances the validity of the adjusted prevalence estimates.

## METHODS

The methods and analysis were not pre‐registered and so the results should be considered exploratory.

### Defining the ‘hidden population’

Using previous definitions and categorisations, the ‘hidden population’ for household surveys was defined as people from or living in [7, 8]:

1. Communal establishments, which refer to places where individuals live or stay collectively outside of the context of a private household. These establishments are characterised by shared facilities and services that support the residents. This category can be further broken down into:
  - Care and residential homes (for the elderly and those with a disability) are facilities for individuals who require assistance with daily living activities.
  - Long‐stay hospitals (including mental health wards) are healthcare facilities where patients with severe or chronic medical conditions, including mental health disorders, receive specialised care over an extended period.
  - Student halls of residence are accommodation provided by educational institutions, typically universities and boarding schools, for their students.
  - Prisons are secure facilities where individuals convicted of crimes serve their sentences as part of the criminal justice system.
  - Hostels for people experiencing homelessness are facilities that provide shelter and basic amenities for individuals experiencing homelessness.
2. Immigration detention centres, which are facilities where individuals awaiting immigration decisions or facing deportation from a country are detained.
3. Gypsy, Roma and Travelling Communities, these communities consist of ethnic groups with nomadic or semi‐nomadic lifestyles. They may live in traditional caravans, trailers, or other temporary dwellings and often maintain distinct cultural practices and traditions.
4. Bed and breakfasts and unsupported temporary accommodation, which offer short‐term accommodations, typically consisting of private bedrooms and shared facilities such as bathrooms and dining areas for those who are experiencing homelessness.
5. Sleeping rough is a term which refers to individuals who sleep in outdoor locations, such as streets, parks, or abandoned buildings, without access to safe and secure housing.
6. Sofa surfing is a term which refers to individuals (sofa surfers) who temporarily stay with friends, family members or acquaintances (referred to as hosts) rather than having a stable and permanent place to live.

### Estimating the size of the ‘hidden population’ and smoking rates

There are no standards for estimating the ‘hidden population’ size, only suggested methods, which include those based on:

1. Independent samples (e.g. multiplier and capture‐recapture): the multiplier method involves using data from two or more independent sources to estimate the size of a hidden population. For example, if a certain percentage of a hidden population is known to have accessed a specific service, and the total number of service users is known, this data can be used to estimate the total hidden population size. Capture‐recapture method involves multiple rounds of sampling and uses the overlap between samples to estimate the total population size.
2. Social networks (e.g. snowball sampling, network scale‐up method and respondent driven sampling): snowballing method begins with a small number of known individuals in the ‘hidden population’ who are asked to recruit further members from their social network. The network scale‐up method estimates the size of a hidden population by leveraging social network data, while respondent driven sampling is a more refined form of snowball sampling that includes statistical adjustments to account for the non‐random nature of the sampling process.
3. Population counting (e.g. Delphi method with experts and mapping locations): the Delphi method uses a structured process where a panel of experts provides estimates based on their knowledge and experience. The estimates are revised over multiple rounds to converge on a consensus. Mapping location involves geographically mapping the locations where members of the hidden population are likely to be found. It can include direct observations, counts, or using geographic information systems to estimate population sizes.
4. Official reports (e.g. workbook method): this method compiles data from various official sources, such as health records, surveys and administrative data, to estimate the size of a ‘hidden population’. It allows researchers to assess the reliability of the data through bias assessments and cross‐validation with other data sources.

For a review of these methods, see Xu *et al*. [12]. The workbook method, which has previously been used to estimate the number of people living with HIV/AIDS [13], was used here to estimate the size of the ‘hidden population’ in the United Kingdom. This method is appropriate as reliable data can be readily retrieved on the rates of individuals in the sub‐populations of interest. The workbook method also enables researchers to conduct bias assessments to evaluate the reliability of the data sources used for estimation.

For this study, the first step was to collect data from government publications, academic studies and official reports that provide information on the characteristics and rates of individuals within the sub‐populations of interest. These sources of data were then combined to give an overall estimate of the size of the ‘hidden population’ in the United Kingdom. The ‘Risk of Bias (RoB) Tool for Prevalence Studies’ was used to estimate the overall risk of bias from the data used to estimate the prevalence of the ‘hidden population’ [14]. The estimates were then refined to reflect those over the age of 18+ years, (APS only collects data on smoking prevalence among this age group). The same method was then used to estimate the rates of smoking within the ‘hidden population’.

### Calculating the impact of excluding the ‘hidden population’

The APS 2022 [3] estimate of smoking prevalence was used as the baseline prevalence. The prevalence rates from the APS were then adjusted to include the additional smokers identified within the ‘hidden population’. Following the adjustment, the coverage error was calculated. This error represents the disparity between the smoking prevalence rates observed in the APS and the adjusted rates that account for the ‘hidden population’. To quantify the impact of the coverage error on smoking prevalence estimates, a correction factor was derived. This factor serves as a metric to adjust future smoking prevalence statistics to account for the previously excluded ‘hidden population’. The correction factor was calculated by determining the ratio of the adjusted smoking prevalence rate (including the ‘hidden population’) to the original APS smoking prevalence rate. This ratio indicates the extent to which the smoking prevalence rates might have been deflated because of the exclusion of the ‘hidden population’.

To assess the robustness of the estimates several sensitivity analyses were conducted by varying the size and smoking prevalence of the hidden population. In the first scenario, we reduced the size of the hidden population by 50%. In the second scenario, we maintained the hidden population size, but reduced their smoking prevalence by 50%. A 50% reduction represents a significant deviation from our initial estimate, allowing us to explore the sensitivity of our results to substantial changes in population size and smoking prevalence. We also assessed under what scenario the population size and smoking prevalence would need to be to have no impact on prevalence estimates (i.e. a correction factor of 1).

## RESULTS

### What is the size of the ‘hidden population’ age 18+ years?

The size of each of the sub‐categories of the ‘hidden population’ is estimated below.

#### Communal establishments

The 2021 census in England and Wales estimated that 1 042 000 individuals (1.7% of the population) lived in communal establishments. This included those in boarding schools, prisons, military bases, hospitals, care homes, student halls of residence and hotels [15]. It is estimated from the 2011 Census that a further 92 000 people in Scotland and 33 524 people in Northern Ireland live in communal establishments [16, 17]. This gives a total for the United Kingdom of approximately 1.2 million individuals. There are some limitations with this estimate. For example, it does not include sheltered accommodation, serviced apartments, and nurses' accommodation. Moreover, although attempts are made to collect data directly from participants, sometimes proxies were used, which may result in inaccuracies or omissions.

#### Immigration detention centres

In 2021, approximately 24 500 people entered immigration detention in the United Kingdom [18]. Data on those entering detention are counts of the number of occurrences of people entering detention rather than the number of unique individuals. Therefore, an individual who enters detention twice would be counted twice in the data. The number also reflects the overall flow of people into the immigration detention system, not just those in immigration detention centres (e.g. short‐term holding facilities), although these are the primary type of facilities. Immigration removal centres also have different recording practices and some individuals are detained for short periods without proper documentation.

#### Gypsy, Roma and Traveling Communities

Given the lack of available data for estimating the size of the Gypsy, Roma and Traveling Communities, data were constrained to those living in caravan sites. The total number of traveller caravans in England in July 2022 was 25 653 [19] and in Wales in January 2021 was 1065 caravans [20]. With an average of 2.4 persons per caravan [21], this equates to approximately 64 000 individuals. In Scotland, there are 613 public and private pitches, in addition to 406 discreet locations that were unauthorised. This equates to approximately 2400 individuals. Data from Northern Ireland estimates that there are 1628 travellers of which 9% live in permanent/serviced sites, which is approximately 147 individuals. Assuming a similar ratio of public and private pitches to discreet locations as in Scotland, this gives a total of approximately 368 individuals [22]. Combined this is approximately 67 000 individuals in the United Kingdom.

This estimate likely underestimates the true population because of several biases. First, it relies solely on individuals living in caravan sites, excluding those who are fully nomadic or reside in non‐permanent or unauthorised locations, which are difficult to capture in official data. The average family size of 2.4 persons per caravan may not reflect the diversity in family structures. Moreover, the assumption that the ratio of public/private pitches to unauthorised sites in Northern Ireland mirrors that of Scotland may not be valid.

#### Bed and breakfasts and other types of unsupported temporary accommodation

There were 94 870 households in temporary accommodation in England at the end of June 2022 [23]. The number of households currently housed in unsuitable temporary accommodation in Scotland is estimated at 2070 [24], 7498 households in Wales [25] and 3596 Northern Ireland [26]. This sums to 108 034 households in the United Kingdom. Assuming an average household size in the United Kingdom of 2.4 this gives an estimated 259 000 individuals in temporary accommodation [15].

The assumption of an average household size of 2.4 may also not reflect the diversity of family structures across different regions or types of accommodation, leading to an over‐ or under‐estimation of the actual number of individuals. Regional differences in the provision of temporary accommodation and varying definitions of what constitutes ‘temporary’ accommodation could also affect the comparability of these figures across the United Kingdom.

#### People sleeping rough

In September 2022, 6631 individuals were estimated to be homeless and sleeping rough in England over a 1‐month period [27]. An additional 405 rough sleepers were estimated in Wales in 2020 (over a 2‐week period) and 2438 households in Scotland (over a 3‐month period) [28, 29]. There is no formal census of the number of people sleeping rough in Northern Ireland. However, estimates are available from the homelessness monitor, which suggests approximately 250 individuals in Northern Ireland sleeping rough on a single night in 2009 [30]. Extrapolating this to a year is difficult because of the seasonal nature of experiencing homelessness. Rough sleeping is also very transient, with individuals moving in and out of homelessness. Therefore, the number of rough sleepers may not significantly change week to week. A conservative approach is to use the longest sampling period available, here 3 months, to standardise estimates:

- England: 6631 × 3 = 19 893 individuals over a 3‐month period
- Wales: 405 × 6 = 2430 individuals over a 3‐month period
- Scotland: 2438 individuals over a 3‐month period
- Northern Ireland, 250 × 84 = 21 000 individuals over a 3‐month period
- United Kingdom = 45 761

To check that these estimates are reasonable based on population size, a population ratio approach was used with Scotland, as the country with the best estimate of rough sleeping, as the reference:

- Population in England is approximately 56 million, which is a ratio of 10.37 of the population in Scotland (~5.4 million). On this basis, we would expect the 3‐month estimate for England to be in the region of 25 282 individuals (2438 × 10.37).
- Population in Wales is approximately 3.2 million, which is a ratio of 0.59 of the population in Scotland (~5.4 million). On this basis, we would expect the 3‐month estimate for Wales to be in the region of 1438 individuals (2438 × 0.59).
- Population in Northern Ireland is approximately 1.9 million, which is a ratio of 0.35 of the population in Scotland (~5.4 million). On this basis, we would expect the 3‐month estimate for Northern Ireland to be in the region of 853 individuals (2438 × 0.35).
- United Kingdom = 30 011

These two approaches give estimates of 45 761 and 30 011 for the United Kingdom with an average of approximately 38 000 individuals.

This could be an overestimate or underestimate because of limitations of the data. Rough sleepers who are transient, hidden or in non‐visible locations are unlikely to be captured. Moreover, data collection methods and definitions of ‘rough sleeping’ vary between regions, which can lead to inconsistencies in the data.

#### Sofa surfers

In 2018 to 2019, 2% of households in England had someone living with them in the last 12 months who would otherwise be homeless (i.e. ‘sofa surfing’) [31]. Given an estimated 28.1 million households in the United Kingdom, this would suggest approximately 562 000 sofa surfers. England and Scotland both include measures relating to sofa surfing in their statutory homelessness statistics, which can be used to validate this estimate. This is achieved by asking questions about households' accommodation type on their homeless application in England and asking households the property type from which they became homeless in Scotland. Between April 2021 to March 2022, 110 792 individuals were reported to be living with friends at the time they made their homeless application in England and Scotland [32]. Although Northern Ireland and Wales collect similar information about types of living situations, they do not include types of accommodation that could reflect sofa surfing. Therefore, the overall estimate can be calculated using a ratio approach:

- Population in England and Scotland is approximately 61.4 million. The population in Wales is approximately 3.2 million, which is a ratio of 0.052. On this basis, we would expect there to be 0.052 × 110 792 = 5761 sofa surfers in Wales.
- Population in Northern Ireland is approximately 1.9 million, which is a ratio of 0.031 of the population in Scotland and England (~61.4 million). On this basis, we would expect there to be 0.031 × 110 792 = 3438 sofa surfers in Northern Ireland.

This gives a total of approximately 120 000 sofa surfers in the United Kingdom. Comparing this figure with the estimated number of sofa surfers based on household survey data suggests a significant portion of those who are sofa surfing may not be captured in formal homelessness statistics. Moreover, it is important to note that this number reflects those who have formally approached authorities for homelessness support and may still not capture all individuals who are sofa surfing informally or without seeking official assistance. Therefore, the higher number of 562 000 was used.

Across all these sub‐categories, it is estimated that the ‘hidden population’ in the United Kingdom is approximately 2.2 million (see Table 1). However, this estimate may under‐ or over‐estimate the true prevalence given biases in data collection. These biases are described briefly in Table 2 below. The lowest risk of bias comes from the estimate of communal establishments, while the highest bias is from the estimate of the number of rough sleepers and Gypsy, Roma and Traveling Communities.

**TABLE 1:** Estimate of the size of the ‘hidden population’ in the United Kingdom.

Hidden population	Estimated size
Communal establishments (care and residential homes, long‐stay hospitals, students in halls of residence, prisons, hostels for homeless)	1 200 000
Immigration removal centres	25 000
Gypsy, Roma and Traveling communities	67 000
Bed and breakfasts and unsupported temporary accommodation	259 000
People sleeping rough	38 000
Sofa surfers	562 000
Total	2 151 000

**TABLE 2:** Bias assessment for prevalence estimates of the ‘hidden population’ (using the RoB tool for prevalence studies).

	Data sources	Description	External validity					Internal validity					Overall risk of bias^a^
			Was the target population representative of the national population?	Was the sampling frame accurate?	Was the sample selected randomly, or was a census used?	Was non‐response bias minimal?	Were data collected directly from subjects?	Was the case definition appropriate?^b^	Was the study instrument valid and reliable?	Was the same data collection method used for all?	Was the prevalence period appropriate?^c^	Were the numerator and denominator correct?	
Communal establishments	Census data across countries [15, 16, 17]	The data provide a highly reliable sample of the population, although certain categories, such as nurses' accommodation, were excluded from the survey. It achieves an ~97% response rate. Data collection combines direct self‐reporting with informant reporting on current living arrangements. Definitions are consistent across countries, ensuring validity and reliability of the measures. However, data were only available for Scotland and Northern Ireland for 2011, with data for England and Wales available only in 2021.	Yes	Yes	Yes	Yes	Partially	Yes	Yes	Yes	Yes	Yes	Low risk
Immigration removal centres	Home Office data [18]	This data focuses primarily on individuals entering immigration removal centres and does not include those in other types of immigration detention facilities, such as short‐term holding facilities. Some individuals may be detained or released without proper documentation, leading to potential gaps in reporting. Practices for recording and reporting data can vary between facilities. The data are measured over a 1‐year period, and individuals who enter detention multiple times within this timeframe are counted repeatedly.	No	Yes	Yes	No	Yes	Yes	No	No	Yes	No	Moderate risk
Gypsy, Roma and Traveling communities	Caravan site data [19, 20, 21, 22]	The data are based on non‐random samples of caravan sites, which may overlook nomadic individuals or those living in unauthorised sites. Collection methods varied, and the data were recorded over a 1‐y period. Estimates were calculated based on the average no. of residents per caravan, which introduces potential variability.	No	No	No	No	No	No	No	No	Yes	No	High risk
Bed and breakfasts/temporary accommodation	Local authority data [23, 24, 25, 26]	Data collection methods and reporting practices vary significantly across local authorities. Estimates are typically based on the average no. of residents per household and definitions of ‘temporary’ accommodation differ by region. The data were collected at a single point in time.	Yes	Yes	Yes	No	No	Yes	No	No	Yes	No	Moderate risk
People sleeping rough	Homeless charity and local authority data [27, 28, 29, 30]	A significant portion of rough sleepers do not engage with services or are hard to reach through point‐in‐time collection methods. Additionally, there is variability in the time periods assessed, ranging from a single night to monthly evaluations. Definitions of ‘rough sleeping’ and data collection methods vary between regions.	No	No	No	No	No	No	No	No	No	No	High risk
Sofa surfers	English Housing and Homelessness Application data [31, 32]	These data pertain only to England and Scotland. The English Housing Survey has a low response rate of 33% and relies on proxies for certain metrics. Homelessness Application data, on the other hand, is limited to individuals who actively make an application. Both datasets are measured over a 1‐y period.	Yes	Yes	No	No	Yes	No	Yes	Yes	Yes	No	Moderate risk

^a^ Low risk = further data is very unlikely to change our confidence in the estimate; Moderate risk = further research is likely to have an important impact on our confidence in the estimate and may change the estimate; High risk = further research is very likely to have an important impact on our confidence in the estimate and is likely to change the estimate.

^b^ A good case definition accurately reflects the living situations you intend to measure.

^c^ A 1‐year prevalence period was considered sufficient to account for most people's accommodation choices, as it captures the diversity of housing situations while balancing the risk of bias because of seasonality or temporary living arrangements. For accommodation types being studied, which change rapidly (such as temporary housing), a shorter prevalence period (e.g. 3–6 months) was considered appropriate. For more stable housing types, current living arrangements were deemed to accurately reflect long‐term patterns.

The above estimates are for the entire population. Eighteen is the legal age of the sale of Tobacco in the United Kingdom. Seventy‐nine percent of the population in the United Kingdom are over the age of 18 [33]. The percentage of the ‘hidden population’ over 18 is likely to be significantly higher because of greater care provisions for children. The estimate ‘hidden population’ age 18+ years in the United Kingdom is given in Table 3 below at 1.9 million.

**TABLE 3:** Estimate of the size of the ‘hidden population’ in the UK age 18+ years.

Hidden population	Data	Estimated size
Communal establishments (care and residential homes, long‐stay hospitals, students in halls of residence, prisons, hostels for homeless)	~95% of those living in communal establishments in England and Wales are adults [34]. This would give an estimated number of adults of (1 200 000 × 0.95) in the UK.	1 100 000
Immigration removal centres	~97% of people entering immigration detention in the UK are age 18+ y [18]. This would give an estimated number of adults of (25 000 × 0.97) in the UK.	24 000
Gypsy, Roma and Traveling communities	Data on numbers of adult and child travellers in caravan sites are not analysed at a population level [34]. Local surveys suggest that ~54% to ~74% (average of 64%) of residents in traveller sites are adults [35, 36]. This would give an estimated number of adults of (67 000 × 0.64) in the UK.	43 000
Bed and breakfasts and unsupported temporary accommodation	~63% of all households in temporary accommodation in England had at least 1 child [37]. Given the average number of children per household is 1.92, this would suggest ~131 k children in temporary accommodation (*n* = 68 100 households with children in temporary accommodation × 1.92) and, therefore, 50% of the total number estimated to be in temporary accommodation [130,678/(108,034*2.4)]. This would give an estimated number of adults of (259 000 × 0.50) in the UK.	130 000
People sleeping rough	Under 18 y who are homeless should be provided with accommodation by Children's Services. Official statistics in England and Wales indicate that there were no people under the age of 18 y found sleeping rough in autumn 2021. Local estimates in London suggest that only ~0.2% of those sleeping rough are under the age of 18 [27, 38]. Therefore, all are assumed to be 18+ y.	38 000
Sofa surfers	Under 18 y who are homeless should be provided with accommodation by Children's Services. Therefore, all are assumed to be 18+ y .	562 000
Total		1 897 000

Abbreviation: UK, United Kingdom.

The estimated total United Kingdom usual resident population in mid‐year 2022 over the age of 18 was 52.3 million [33]. Therefore, the total population including the estimated ‘hidden population’ of those ages 18+ years is:

$$\text{Estimate}\;N_{\text{total population aged}\;18+}=52\;300\;000+1\;897\;000=54\;197\;000.$$

The estimated percentage of the population who are hidden is:

$${\text{Estimate percentage}}_{\text{hidden}}=\left(\frac{n_{\text{hidden aged}\;18+}}{n_{\text{population aged}\;18+}}\right)\times 100=\left(\frac{1\;897\;000}{54\;197\;000}\right)\times 100=3.5\%.$$

### What is the prevalence of tobacco use?

#### Communal establishments

No estimates are available for all communal establishments. Therefore, this was broken down into the following separate categorises:

- Care and residential homes (for the elderly and those with a disability): estimates suggest that smoking prevalence among those with a disability may be approximately 20% to 30% [39], but this likely varies by age. For example, smoking prevalence has been estimated to reach approximately 70% for young people accommodated in residential care‐homes [39]. Data is lacking for older adults, but in the general population in England age 65+, we know that the prevalence of smoking approximately 8% [40].
- Long‐stay hospitals (including mental health wards): smoking bans are implemented across most (~80%) mental health trusts in England and Wales, but not Scotland [34]. Where bans on inpatient mental health wards are not in place, smoking prevalence is approximately 50% [35].
- Students in halls of residence: prevalence among students living in halls of residence is similar to that of the population age 18 to 24 years (~13%) [36]. Most university campuses in the United Kingdom have implemented smoking bans.
- Prisons: smoking in prisons is estimated to be approximately 80% despite smoking bans indoors [41].
- Hostels for People Experiencing Homelessness: smoking prevalence among people experiencing homelessness who reside in hostels can be high because of factors such as social disadvantage, mental health issues and addiction. Estimates suggest that smoking prevalence among people experiencing homelessness may be approximately 70% to 80%, but this can vary depending on the population and location [42].

Ratios of those living in these various communal establishments are not available. Therefore, a weighted average cannot be calculated. Using the highest and lowest prevalence rates within each setting, a range that encompasses the potential variability was derived:

$$\left(8\%+50\%+13\%+80\%+70\%\right)/5=44.2\%,$$

$$\left(70\%+50\%+13\%+80\%+80\%\right)/5=58.6\%.$$

#### Immigration removal centres

Smoking is allowed in outdoor spaces, but not within the removal centres in the United Kingdom. No data are available on smoking status within immigration removal centres, and so migrant smoking prevalence figures from the APS are used as a proxy. Data suggests that whereas migrant women are less likely to smoke than UK‐born women (~10%), the opposite is true for men (~19%) [43].

#### Travellers in caravan sites

Data from 2014 pooled across the Integrated Household Survey and the general practitioner (GP) Patient Survey, suggests that smoking rates are approximately 46% to 49% [44].

#### People experiencing homelessness

No estimates are available separately for bed and breakfasts and unsupported temporary accommodation, people sleeping rough and sofa surfers. Therefore, these were combined into one category. A recent systematic review assessed smoking prevalence in populations experiencing homelessness, reporting a rate of between 57% and 82%, with an average of 70% [42]. This is supported by a more recent estimate from the Homeless Health Needs Audit of approximately 80% [45].

Table 4 summarises the risks associated with smoking estimates in each group, reflecting the potential biases in study design, sampling, reporting and measurement. The overall risk of bias is moderate for most groups.

**TABLE 4:** Bias assessment for smoking prevalence estimates of the ‘hidden population’ (using the RoB tool for prevalence studies).

	Data sources	Description	External validity				Internal validity						Overall risk of study bias^c^
			Was the target population representative of the national population?	Was the sampling frame accurate?	Was the sample selected randomly, or was a census used?	Was non‐response bias minimal?	Were data collected directly from subjects?	Was the case definition appropriate?^a^	Was the study instrument valid and reliable?	Was the same data collection method used for all?	Was the prevalence period appropriate?^b^	Were the numerator and denominator correct?	
Communal establishments	No overall estimate is available for each type and so estimates were combined.	The data were derived from non‐representative and non‐random samples, with several studies experiencing high non‐response bias. Data collection methods combined direct self‐reporting and informant reporting. Additionally, differences in recording practices within and between studies, as well as the use of varying data collection methods, further impact consistency. All studies asked participants directly whether they were currently smoking.	No	No	No	No	Partially	No	No	No	Yes	Yes	High risk
Immigration removal centres	Migration Observatory using data from the Annual Population Survey [43]	The migrant population is used as a proxy in the study, with data collected through random sampling. However, response rates may be as low as 50% [43]. Data are generally collected directly from individuals using standardised questions on current smoking.	No	No	Yes	No	Yes	Yes	Yes	Yes	Yes	Yes.	Moderate
Travellers in caravan sites	Integrated Household Survey and the GP Patient Survey [44]	The study focuses on Gypsy and Irish travellers who are registered with a GP, using a random sample for data collection. However, response rates may be as low as 27% [46]. Data are generally collected directly from individuals using standardized questions on smoking, with all participants asked if they were currently smoking, ensuring consistency in this key measure.	No	No	Yes	No	Yes	Yes	Yes	Yes	Yes	Yes.	Moderate
People experiencing homelessness	Systematic review and Homeless Health Needs Audit [42]	The data generally come from individuals visiting homeless centres or healthcare facilities. Several studies experienced high non‐response bias. Data collection relied on a combination of direct self‐reporting and informant reporting. Additionally, different recording practices were used between studies. All studies asked participants whether they were currently smoking.	No	No	No	No	Partially	No	No	No	Yes	Yes	Moderate

Abbreviation: GP, general practitioner.

^a^ A good case definition accurately captures smoking status, ensuring that it reflects individuals' true smoking behaviour.

^b^ Current smoking status is an appropriate prevalence period as it provides a clear snapshot of smoking behaviour in the population at a specific point‐in‐time.

^c^ Low risk = further data is very unlikely to change our confidence in the estimate; Moderate risk = further research is likely to have an important impact on our confidence in the estimate and may change the estimate; High risk = further research is very likely to have an important impact on our confidence in the estimate and is likely to change the estimate.

The estimated total proportion of the ‘hidden population’ who smoke is given in Table 5. Two estimates are given based on the upper and lower ranges of smoking prevalence in the ‘hidden population’ of interest from previous research. These estimates were calculated by weighting the proportions derived from the previous literature by the contribution of the group of interest to the total population of hidden individuals. Using the population estimate this would suggest that the number of smokers in the ‘hidden population’ is between approximately *n* = 1 093 127 and *n* = 1 255 093.

**TABLE 5:** Estimated smoking prevalence among the ‘hidden population’ in the United Kingdom.

Population	Weighting	Upper smoking proportion estimate	Lower smoking proportion estimate	Weighted upper smoking proportion estimate	Weighted lower smoking proportion estimate
Communal establishments	0.580	0.586	0.442	0.340	0.256
Immigration removal centres	0.013	0.190	0.100	0.002	0.001
Travellers in caravan sites	0.029	0.490	0.460	0.011	0.011
Bed and breakfasts/temporary accommodation	0.069	0.80	0.80	0.055	0.055
People sleeping rough	0.020	0.80	0.80	0.016	0.016
Sofa surfers	0.296	0.80	0.80	0.237	0.237
Total estimate:				0.662	0.576

Abbreviation: UK, United Kingdom.

### What is the impact of ignoring the ‘hidden population’?

The APS estimated that in the United Kingdom in 2022, 12.9% of people age 18 years and over living in households smoked cigarettes, which equates to approximately seven million people in the population [52 300 000 × 0.129 = 6 746 700]. Based on the estimation of the size of the ‘hidden population’ we can come to two new estimates of the size of the total smoking population.

$$\begin{array}{l} \text{Upper}\;\text{estimate}\;n_{\text{smokers}\;\text{aged}\;18+}=6\;746\;700+1\;255\;093=8\;001\;793\\ \text{Lower}\;\text{estimate}\;n_{\text{smokers}\;\text{aged}\;18+}=6\;746\;700+1\;093\;127=7\;839\;827\\ \text{Upper}\;\text{estimate}\;\text{smoking}\;\text{prevalence}=\left(\frac{n_{\text{smokers}\;\text{aged}\;18+}}{n_{\text{population}\;\text{aged}\;18+}}\right)\times 100\\ \;=\left(\frac{8\;001\;793}{54\;197\;000}\right)\times 100=14.8\%\\ \text{Lower}\;\text{estimate}\;\text{smoking}\;\text{prevalence}=\left(\frac{n_{\text{smokers}\;\text{aged}\;18+}}{n_{\text{population}\;\text{aged}\;18+}}\right)\times 100\\ \;=\left(\frac{7\;839\;827}{54\;197\;000}\right)\times 100=14.5\% \end{array}$$

This suggests an absolute percentage increase in the prevalence statistics of between 1.6% and 1.9% in 2022 (coverage bias). The correction factor, estimated as a ratio of the APS prevalence statistic for the United Kingdom compared to the estimate including the ‘hidden population’ is 1.12 to 1.14.

Table 6 shows the estimates of smoking prevalence for the United Kingdom from the APS stratified by country, along with the 95% confidence interval and inflated estimate using the correction factor of 1.14. The APS estimate for smoking prevalence in England, Wales, Scotland and Northern Ireland shows no overlap with the inflated estimates confidence intervals. This suggests a significant discrepancy, indicating that the APS estimate might be underestimating smoking prevalence. However, when the correction factor of 1.12 is applied, the APS estimates for regions other than England have confidence intervals that overlap with the confidence intervals of the inflated estimates. This implies that while there might still be differences between the APS and inflated estimates, these differences are not as pronounced. One possible reason for this could be differences in sample size across regions. Larger sample sizes typically lead to more precise estimates that are closer to the true population parameters (see Table S1).

**TABLE 6:** Smoking prevalence across the UK estimated by the APS and new estimates with the correction factor 1.14 applied.

Countries	APS prevalence	APS prevalence lower 95% CI	APS prevalence upper 95% CI	APS prevalence with correction factor	APS prevalence lower 95% CI with correction factor	APS prevalence upper 95% CI with correction factor
UK	12.9			14.7		
England	12.7	12.3	13.0	14.5	14.0	14.8
Wales	14.1	13.2	14.9	16.1	15.0	17.0
Scotland	13.9	13.0	15.6	15.8	14.8	17.8
Northern Ireland	14.0	13.0	14.9	16.0	14.8	17.0

*Note*: CI are not given for the UK by the APS.

Abbreviations: APS, Annual Population Survey; UK, United Kingdom.

Sensitivity analyses showed that even if the hidden population size was reduced by 50% (*n* = 948 500) with a smoking prevalence of 66.0%, that the overall population smoking prevalence would still be higher at 13.8% with a correction factor of 1.07. Additionally, if the hidden population remained the same, but their smoking prevalence was reduced by 50% (i.e. 33%) the overall smoking prevalence would be 13.6% with a correction factor of 1.05. (see Table S2).

In fact, the hidden population would need to be as small as 60 000, assuming a smoking prevalence of 66%, or smoking prevalence in the hidden population would need to be as low as 14%, assuming the estimated hidden population is correct, for it not to impact on prevalence estimates (i.e. a correction factor of 1).

## DISCUSSION

This study illustrates how household surveys conducted in the United Kingdom may inadvertently underestimate smoking prevalence by a considerable margin. For instance, the smoking prevalence estimates derived from the APS in 2022, initially reported at 12.9%, could potentially be as high as 14.8% when accounting for the ‘hidden population’. This discrepancy translates to a difference of more than one million smokers, highlighting the substantial impact of excluding certain population segments from survey data.

### Policy and public health implications

Underestimating smoking rates has significant implications. First, for governments and healthcare systems like the National Health Service in the United Kingdom, promoting social inclusion and integration is pivotal [47]. Accurate data collection on hidden or marginalised groups is essential for tailoring interventions and services to diverse population needs, enabling informed policy decisions and effective health monitoring [48]. Second, within the United Kingdom context, the challenges posed by the cost‐of‐living and housing crises exacerbate the trends of homelessness, underscoring the urgent need to address these issues as part of public health priorities [49]. Third, addressing health inequalities from a social justice perspective is crucial, because these disparities often result from structural factors that the affected communities have little or no control over. Improved research methods can help rectify these injustices by accurately documenting the needs of marginalised populations [50]. Fourth, effective healthcare planning necessitates precise data on populations affected by specific health issues. The ‘hidden population’, although a minority, often face disproportionate health challenges and either avoid health care services or rely heavily on emergency care services when their health problems become acute [51].

These results show that the inclusion of the ‘hidden population’ in prevalence estimation is crucial for accurate planning and policy formulation, especially given several new funding initiatives have been announced to drive down smoking rates. The UK government has set an ambitious target to achieve a smokefree England by 2030, with a smoking prevalence target of less than 5% for combustible tobacco. It is estimated that achieving this is already delayed by approximately 8 years, from 2030 to 2038, assuming a decline in smoking prevalence of approximately 0.5 percentage points per year [52, 53]. This study would suggest it could be delayed even further until 2042, roughly 12 years later than the target if the true prevalence in 2022 is 14.8% (see Table S3).

### Recommendations and next steps

This study aims to raise awareness about the potential impact of coverage error. Hopefully, this will stimulate interest and research into how we accurately identify and quantifying the ‘hidden population’ of smokers who are not adequately captured in traditional household surveys. Indeed, research is already under way by the ONS and other organisations, including the Department for Levelling Up, Housing and Communities, on how to address the data gaps identified [32]. Looking ahead, it may be useful to consider alternative data sources such as cigarette sales data (although it may not capture illicit use), discarded cigarette packs (although this method has feasibility challenges) and wastewater analysis. These methods could complement household surveys by providing additional insights into smoking behaviours that might be missed through traditional survey methods alone. Routine data collection from secondary care services and third sector (non‐government charity services) on smoking status is also necessary, but to do this funding is required and the data needs to be shared.

This study also advocates for transparency in informing the public about the limitations of household surveys in capturing the full scope of smoking prevalence. The public's statistical literacy has notably improved since the coronavirus disease pandemic, and there is a growing awareness of the complexities involved in data collection methodologies [54]. It is essential to openly address this by clearly stating that household surveys may not fully represent all segments of society.

By acknowledging the populations excluded from these surveys, such as certain demographic groups or those with transient living arrangements, policymakers and researchers can better understand that current prevalence estimates may underestimate the true extent of smoking in the United Kingdom. Furthermore, the challenge of coverage error extends beyond smoking surveys in the United Kingdom; it applies to other household surveys measuring various behaviours and conditions in different countries. Addressing coverage error is a shared challenge globally, requiring collaborative efforts where feasible. Indeed, the ONS is also considering how other countries are capturing the ‘hidden population’ in their data and evidence [32].

Importantly, the goal of this study is not to disrupt established time series or suggest a change in the methods of data collection that have proven valuable in monitoring smoking trends over time. Household surveys have been instrumental in providing consistent and comparable data on smoking prevalence, which is crucial for policy formulation and evaluation of public health interventions. Rather, this study is meant to provide a simple statistical tool in the correction factor that can be applied to historical timeseries to more accurately reflect actual smoking prevalence.

### Study limitations and advantages

The study serves as an initial exploration into estimating ‘hidden populations’ and understanding the impact of their exclusion from population surveys. The workbook method was chosen for its practicality, as it uses readily available data from government documents, academic studies and official reports. It therefore serves as an actionable starting point that further research can build on by using alternative methods for triangulation.

This study also offers a pragmatic cost‐effective means by which to adjust prevalence estimates to account for the ‘hidden population’. An alternative approach is to enhance survey coverage by using mixed modes of data collection, such as combining household surveys with action‐based sampling among those using relevant homeless services [55]. Including communal establishments in household surveys, leveraging census weighting and adapting surveys to include temporary household residents are also options. However, all these pose challenges for maintaining data integrity over time and are highly intensive. A less intensive option involves collecting information on prior inclusion in a ‘hidden population’, such as asking respondents about past experiences like sofa surfing. Responses from individuals with such histories can be weighted more heavily to better reflect the ‘hidden population’. This, however, would require complex weighting with a risk of overcompensation. Ensuring that the weighting appropriately reflects the proportion of the hidden population without exaggerating their numbers is difficult and requires careful calibration and validation.

However, there are also limitations inherent in estimating a correction factor. Economic conditions, public policies and societal attitudes can influence the size and composition of the ‘hidden population’, meaning that a correction factor is unlikely to be static. Therefore, it may be necessary to continually update this over time. This study also used the workbook method, which relies on official reports, and these reports may not provide accurate estimates of the ‘hidden population’ and smoking prevalence within them. Additionally, variations in how the ‘hidden population’ are defined and captured across different nations of the United Kingdom pose challenges to consistency in measurement and reporting. In cases where data needed to be combined across different timepoints, there is a risk of temporal biases, especially if there have been significant changes in population dynamics, policies or methodologies over time.

Additionally, relying on assumptions to fill data gaps or to extrapolate estimates introduces uncertainties that can affect the accuracy of the final estimates. The challenges were greatest when estimating the number of rough sleepers and Gypsy, Roma and Traveling Communities. Furthermore, this study assumes stability in ‘hidden population’ size over time, failing to account for their transient nature and movement in and out of the general population. Moreover, this study does not differentiate between vulnerable individuals within the ‘hidden population’, such as those sleeping rough and those who are not generally vulnerable, like students in halls of residence. This distinction is vital for addressing health inequalities, as smoking rates may vary significantly between these groups. Recent data also suggests that the ‘hidden population’ living in poverty may in fact be growing [49].

## CONCLUSIONS

This study highlights that household surveys excluding the ‘hidden population’ may substantially underestimate smoking rates, potentially impacting policy formulation and healthcare planning. Based on the estimated correction factor, and assuming smoking continues to decline at current rates, achieving the UK government's target of 5% would likely be delayed by over a decade.

## AUTHOR CONTRIBUTIONS

**Emma Beard:** Conceptualization; methodology; data curation; writing—original draft; writing—review and editing. **Jamie Brown**, **Sharon Cox** and **Lion Shahab:** Methodology; writing—review and editing.

## DECLARATION OF INTERESTS

This report is part of the UCL 2022/2023 Health and the Public Impact Fellowship awarded to E.B. Cancer Research UK provides salary support for SC (PRCRPG‐Nov21/100002). There are no other conflicts of interest.

## Supporting information


**Table S1:** Smoking prevalence across the UK estimated by the APS and new estimates with the correction factor 1.12 applied.
**Table S2:** estimated prevalence when changing the hidden population size and smoking prevalence for the hidden population.
**Table S3:** estimated prevalence and attainment of the 2030 target prevalence below 5% when starting at the APS estimated target and APS inflated estimate.

## Data Availability

Data are included in the manuscript.
