# Supplementary material for: How much does the absence of the ‘hidden population’ from United Kingdom household surveys underestimate smoking prevalence?
Source: Addiction. 2025 May 2;120(8):1582–600. doi: 10.1111/add.70071 (PMC12215282; doi:10.1111/add.70071)
Supplement: Supplementary file 1 — Table S1: Smoking prevalence across the UK estimated by the APS and new estimates with the correction factor 1.12 applied. Table S2: estimated prevalence when changing the hidden population size and smoking prevalence for the hidden population. Table S3: estimated prevalence and attainment of the 2030 target prevalence below 5% when starting at the APS estimated target and APS inflated estimate. [file ADD-120-1582-s001.docx]

**Supplementary Table 1:** Smoking prevalence across the UK estimated by the APS and new estimates with the correction factor 1.12 applied

| Countries | APS prevalence | APS prevalence Lower 95%CI | APS prevalence Upper 95%CI | APS prevalence with correction factor | APS prevalence Lower 95%CI with correction factor | APS prevalence Upper 95%CI with correction factor |
| --- | --- | --- | --- | --- | --- | --- |
| UK | 12.9 |  |  | 14.5 |  |  |
| England | 12.7 | 12.3 | 13.0 | 14.2 | 13.8 | 14.6 |
| Wales | 14.1 | 13.2 | 14.9 | 15.8 | 14.8 | 16.7 |
| Scotland | 13.9 | 13.0 | 15.6 | 15.6 | 14.6 | 17.5 |
| Northern Ireland | 14.0 | 13.0 | 14.9 | 15.7 | 14.6 | 16.7 |

Note: confidence intervals are not given for the UK by the APS

**Supplementary Table 2:** estimated prevalence when changing the hidden population size and smoking prevalence for the hidden population

| Total UK usual resident population (N) | Hidden population (n) | Total estimate population (N + n) | Smoking prevalence in the UK population (P) | Smoking prevalence in the hidden population (P)h | Number of smokers in the UK population (P x N) | Number of smokers in the hidden population (Ph x n) | Total number of smokers (P x N)+(Ph x n) | Estimated smoking prevalence) | Correction factor (P:Y) |
| --- | --- | --- | --- | --- | --- | --- | --- | --- | --- |
| 52,300,000 | 1,897,000 | 54,197,000 | 0.129 | 0.662 | 6,746,700 | 1,255,093 | 8,001,793 | 0.148 | 1.14 |
| 52,300,000 | 1,897,000 | 54,197,000 | 0.129 | 0.576 | 6,746,700 | 1,093,127 | 7,839,827 | 0.145 | 1.12 |
| 52,300,000 | 948,500 | 53,248,500 | 0.129 | 0.662 | 6,746,700 | 627,547 | 7,374,247 | 0.138 | 1.07 |
| 52,300,000 | 1,897,000 | 54,197,000 | 0.129 | 0.331 | 6,746,700 | 627,547 | 7,374,247 | 0.136 | 1.05 |

**Supplementary Table 3:** estimated prevalence and attainment of the 2030 target prevalence below 5% when starting at the APS estimated target and APS inflated estimate

| Year | APS estimate | Inflated APS estimate |
| --- | --- | --- |
| 2022 | 12.9 | 14.8 |
| 2023 | 12.4 | 14.3 |
| 2024 | 11.9 | 13.8 |
| 2025 | 11.4 | 13.3 |
| 2026 | 10.9 | 12.8 |
| 2027 | 10.4 | 12.3 |
| 2028 | 9.9 | 11.8 |
| 2029 | 9.4 | 11.3 |
| 2030 | 8.9 | 10.8 |
| 2031 | 8.4 | 10.3 |
| 2032 | 7.9 | 9.8 |
| 2033 | 7.4 | 9.3 |
| 2034 | 6.9 | 8.8 |
| 2035 | 6.4 | 8.3 |
| 2036 | 5.9 | 7.8 |
| 2037 | 5.4 | 7.3 |
| 2038 | 4.9 | 6.8 |
| 2039 | 4.4 | 6.3 |
| 2040 | 3.9 | 5.8 |
| 2041 | 3.4 | 5.3 |
| 2042 | 2.9 | 4.8 |
